# Sustainable Development for Film-Induced Tourism: From the Perspective of Value Perception

**DOI:** 10.3389/fpsyg.2022.875084

**Published:** 2022-06-03

**Authors:** Kui Yi, Jing Zhu, Yanqin Zeng, Changqing Xie, Rungting Tu, Jianfei Zhu

**Affiliations:** ^1^School of Business and Trade, Nanchang Institute of Science & Technology, Nanchang, China; ^2^Media Art Research Center, Jiangxi Institute of Fashion Technology, Nanchang, China; ^3^Guangdong University of Finance and Economics, Guangzhou, China; ^4^Department of Art Integration, Daejin University, Pocheon, South Korea; ^5^School of Business, Foshan University, Foshan, China; ^6^College of Management, Shenzhen University, Shenzhen, China; ^7^School of Economics and Management, East China Jiaotong University, Nanchang, China

**Keywords:** film-induced tourism, tourism destination, sustainable development, dynamic mechanism, culture and industry integration

## Abstract

The tourism economy has become a new driving force for economic growth, and film-induced tourism in particular has been widely proven to promote economic and cultural development. Few studies focus on analyzing the inherent characteristics of the economic and cultural effects of film-induced tourism, and the research on the dynamic mechanism of the sustainable development of film-induced tourism is relatively limited. Therefore, from the perspective of the integration of culture and industry, the research explores the dynamic mechanism of sustainable development between film-induced culture and film-induced industry through a questionnaire survey of 1,054 tourism management personnel, combined with quantitative empirical methods. The conclusion shows that the degree of integration of culture and tourism is an important mediating role that affects the dynamic mechanism of sustainable development of film-induced tourism, and the development of film-induced tourism depends on the integration of culture and industry. Constructing a diversified industrial integration model according to local conditions and determining the development path of resource, technology, market, product integration, and administrative management can become the general trend of the future development of film-induced tourism.

## Introduction

As an emerging industry, cultural tourism can make up for the economic difficulties caused by the weak growth of the primary and secondary industries, and replace it as a new driving force for economic growth (Liu et al., 2021b). As recognized by both the academic community and industry, cultural tourism products greatly impact tourism destination development; souvenir, local cuisines, and films/television programs can promote tourism destinations (Liu et al., 2021a,b). Among them, film/television is the most influential form of art in today’s society. Film and television can help potential tourists to have some sensory and emotional cognitions through empathy and vicarious feeling to the tourist destinations mentioned in the films (Kim and Kim, 2018; Pérez García et al., 2021), thereby generating tourism motivation and ultimately promoting tourism behaviors. Film and television, without exception, have integrated commerciality and artistry since birth, and form a unique form of culture (Riley et al., 1998).

Hence, these significant economic effects have been widely investigated by researchers from different perspectives, such as promotion of local brands (Liu et al., 2021a), and changes in aesthetic information dissemination (Kim et al., 2019). With in-depth studies, researchers have identified the profound connotation of the rapid development of film-induced tourism: the extension of the immersive tourism (Marafa et al., 2020), the endowment of modern fashion labels for tourism destinations (Teng and Chen, 2020), and multi-dimensional integrations of modern media technology and traditional entertainment industry.

Culture is the soul of tourism, and tourism is an important carrier of culture. Although the experience of film/television is different from tourism—the former is provided to people by means of image transmission, and the latter is realized by the way of people moving—but the essence is both cultural experience (Syafrini et al., 2020; Senbeto and Hon, 2021). The connotation and the applied research of film-induced tourism reveal the complexity and diversity of the integrations of modern media and traditional entertainment.

The traditional glimmering style sightseeing tour is just a shallow taste, and often cannot make tourists get a deep enjoyment. Film-induced tourism is different, mature film-induced tourism products can bring tourists wholehearted relaxation and enjoyment, and make tourists’ self-worth better reflect. To clarify the inherent characteristics of film-induced tourism, the interactive observation of both the film and television subject and the tourism subject provides a feasible solution. Film/television programs are the expression and substantiveness of culture (Yi et al., 2020). Tourism as an economic carrier is the pattern and standardization of the industry (Yen and Croy, 2016). The development of film-induced tourism relies on the mutual integration of culture and industry.

With the evolution of the world, sustainable development is leading the way in every industry including tourism. The early understanding of sustainable development in the academic community refers to meeting the needs of the current generation without damaging the needs of future generations’ development (Jabareen, 2008; Yi et al., 2021a). Based on this concept, the United Nations has formulated 17 sustainable development goals, proposed new standards for the prosperity and development of the earth, and standardized the assessment methods and indicators for sustainable development (Böhringer and Jochem, 2007; Hacking and Guthrie, 2008; Singh et al., 2009). Since then, the concept of sustainable development has been fully implemented and has gradually become a well-known concept from the perspectives of the environment, economy, and society (Adedoyin et al., 2021; Diep et al., 2021; Zhou et al., 2021). Currently, these three dimensions are identified as the motivations and mechanisms of sustainable development (Steffen et al., 2015; Svensson and Wagner, 2015). Specifically, challenges in sustainable development are vital issues for exploring social and economic development. Economic benefits are the main dynamics of continuous action (Hoogendoorn et al., 2015), with social effects as the main motivation of practice (Williams and Schaefer, 2013), and environmental effects as the basic assurances of all activities (Halme and Korpela, 2014). Hence, sustainable development research help explore the path of the industry development. The dynamic mechanism of sustainable development builds the foundation for the long-term influence of the culture and provides the way for continuous development and expansion of industrial effects (Waheed et al., 2020). At present, to the best of our knowledge, very few studies have investigated the dynamic mechanism of the sustainable development for film-induced tourism. The existing studies which include the sustainable development dynamic mechanism can be divided into three aspects:

(1) The macro sustainable development concept of film-induced tourism (Wen et al., 2018); (2) The sustainable development concept in the exploration of film-induced tourism (Gong and Tung, 2017; Teng, 2021); (3) The micro sustainable development concept of film-induced tourism (Suni and Komppula, 2012). Afterward, most of the studies believe that the dynamic mechanism of sustainable development is affected by its resource development, innovation mode, or artistic attractions. However, these have not yet conducted a quantitative study of the endogenous interactions between culture and industry. Accordingly, we try to fill the research gap; we study the relationship between culture and industry in film-induced tourism through structural equation modeling to promote the sustainable development dynamics brought about the integration of culture and industry.

## Literature Review

### Film-Induced Culture and Tourism Industry

Film-induced culture plays a vital role in the global advertisement system. It is an effective approach for the advertisement of regional values and soft power, and it is a good pathway for cultural output and value proposition (Yi et al., 2020). With the advance of economic development, consumers have broken the restrictions of basic needs spending (Sun et al., 2017, 2021; Du et al., 2020), and the needs for higher-level cultural consumption are becoming increasingly important (Wang et al., 2020a,b; Li et al., 2021). Film-induced culture is by no means limited to entertainment culture, and film-induced products are by no means limited to spiritual and cultural consumer goods (Chen, 2018). Film/television is also a mass media. Film-induced culture has an unprecedented impact on people’s ways of thinking, social cognition, behavioral habits, and values, showing unique cultural tension and becoming an important structure of people’s spiritual life (Misra, 2000; Janssen et al., 2008). Otherwise, as a fast-growing important new tourism trend, film-induced tourism creates connections between characters, places, stories, and tourists, and is inspired to immerse themselves in films to relive film-generated and film-driven emotions (Riley et al., 1998). Essentially, both film and tourism provide an opportunity to relive or experience, see and learn novelties through entertainment and fun (Teng, 2021). Film-induced tourism increases the overall economic effect of tourism industry and establishes the bonds of film and tourism industry. It provides not only pleasure and satisfaction for film-induced tourists, but also adequate and novel learning experience. The latest research trends are moving toward merging or collaborating two fields that already have similar goals.

The integration of film-induced culture spreads information through film-induced programs to “maximize” the effect of tourism cultural brands (Huang and Liu, 2018). The fundamental reason is that the penetration of film-induced culture has driven the transformation and upgrading of tourism consumption (Michael et al., 2020), which in turn makes film-induced culture a resource for tourism development, amplifies the effect of cultural integration in the process of transformation, and further enhances the influence of the tourism industry (Marafa et al., 2020). The establishment of film-induced cities and film-induced bases creates the advantages of film-induced culture agglomeration, and the innovative path of developing film-induced cultural resources oriented by the tourism industry is becoming more and more popular (Ringle, 2018). Cultural resources are further optimized and reorganized, and film-induced culture will gain a series of new integrated development in the promotion of tourism industry model (Xin and Mossig, 2017). On the one hand, the film-induced bases can be used for film/television production, and on the other hand, it is an important place for tourism activities, which truly reflects the integration from products, markets, enterprises, and industries in film-induced tourism industry (Stuckey, 2021). Accordingly, some researchers believe that the establishment of Hollywood Studios in 1963 marked the official beginning of film-induced tourism. Hence, film-induced culture can promote the tourism industry to shape brand culture, integrate useful resources, guide consumer trends, and induce convergence effect for rapid development and innovation (Wu and Lai, 2021). Hence, the following hypotheses are proposed:

*H1a:* The development of film-induced culture is positively related to the growth of the tourism industry.

*H1b:* The cultural development in film-induced tourism is positively correlated to the degree of cultural and tourism integration.

*H1c:* The development of the tourism industry is positively correlated to the degree of culture and tourism integration.

### Culture and Tourism Integration and Sustainable Development

The integration of culture and tourism is not only the objective need for the mutual prosperity of culture and tourism, but also the inevitable trend of the development. The elements compete, cooperate and co-evolve with each other, so that an emerging industry can be formed, and it has experienced “grinding-integration-harmony” of the dynamic development process (Jovicic, 2016; Wang and Yi, 2020). Culture and tourism have a certain basis for mutual benefit and cooperation: for tourism, the integration of cultural-related content helps to acquire extensive knowledge, distant experience, and strong care; for culture, it is conducive to the protection and inheritance of cultural resources, image building, and propagation (Loulanski and Loulanski, 2011; Jørgensen and McKercher, 2019). The integration of culture and tourism is an intimate contact between “poems and dreams,” which better meets people’s diverse needs for a beautiful life (He et al., 2021). However, sustainable development refers to comprehensive and sustainable advancements in ecological, social, and economic aspects. The cognition that based on these three goals can be used to explore the dynamic mechanism for sustainable development of film-induced tourism.

In the dimension of sustainable ecological development, with the advent of the scientific revolution and the industrial revolution, the world is entering a new era. The utilization of resources is not limited to the development of physical resources but is more prone to the rational use of new resources, such as talented person, technology, intelligence, and data (Waheed et al., 2020; Zhang et al., 2020; Li et al., 2021), cultural resources, such as historical culture, red culture, and folk culture, are integrated with tourism resources, such as landscape pastoral, to develop complementary advantages. The maximization of resource utilization has become the key to the sustainable development of the film-induced tourism society, and culture has become a regulator of various innovation factors, which promotes the scientific management of technological and industrial resources (Delai and Takahashi, 2011; Liu et al., 2021b). When transforming and utilizing film-induced cultural resources, do not trample or destroy the ecological environment for tourism development, and comprehensively optimize the tourism environment and tourism routes. Environmentalism and related laws and regulations have begun to pay attention to tourists’ needs (Li et al., 2020). Hence, the further integration of culture and tourism can reflect the transformation of the overall ecological commitment (Zhou et al., 2021), and the resulting human–environment relationship has become a new aspect of sustainable development.

In the dimension of sustainable social development, on the one hand, the improvement of cultural quality of the whole society is a prerequisite for the organic integration of culture and tourism (Tien et al., 2021), with harmonious coexistence becoming the core aspect of economic and cultural development of the new era, tourists and other stakeholders of the film-induced tourism industry begins to focus on human capital development, social recognition, job creation, and health and safety-related issues (Choi and Ng, 2011). With the deepening of research, researchers found that the above-mentioned problems are ideologically attributed to culture and are the society’s force for inducing the sustainable development of industries (Cai and Zhou, 2014). The extension and connotation of tourism need the guidance of tourism culture. Cultural display or visitable production expands the scope of displayable culture, from material to non-material, to the integration of non-material and material, and then to the contemporary creative cultural display, which makes culture continuously “commoditized” (Silberberg, 1995; Marques and Pinho, 2021). At present, many scholars have reached a consensus that the integration of culture and industry can promote the construction of the social community (Jakhar, 2017; Yi et al., 2021b) and promote the relevant members of the society to change their misconduct, thereby strengthening the sustainable development of the film and television industry and the tourism industry.

In the dimension of sustainable economic development, scholars generally agree that economic factors, which refer to the renewable and non-renewable resources invested in the production process, are composed of factors, such as cost, profit, and business development (Mamede and Gomes, 2014; Wagner, 2015). Given the direct impact of economic effect on tourist activities is significant, most researchers directly view economic factors as the main driving force for the sustainable development of film-induced tourism, owing to the direct influence of economic effects on tourists’ tourism activities (Horbach et al., 2013; Hojnik and Ruzzier, 2016). As been defined by researchers, sustainable economic development involves the exploration and innovation of business models, creating market opportunities, the processes of resolving unsustainable environmental and social problems (Schaltegger et al., 2016). When film-induced culture is continuously produced into cultural tourism products, the commercial interests of tourism sales promote the industrialization and gradually form a complete industrial chain-cultural tourism industry. In the studies of film-induced tourism, many researchers view film-induced culture as a resource for creating new business models and market opportunities and regard the integration of film-induced culture with the tourism industry as a solution for unsustainable development problems. In summary, in film-induced tourism, in both the ecological, social, and economic dimensions, the integration of culture and industry will influence the path of sustainable development. Hence, the following hypotheses are proposed:

*H2a:* The degree of integration between film-induced culture and the tourism industry is positively related to the sustainable development of the ecology (human–environment integration).

*H2b:* The degree of integration between film-induced culture and tourism industry is positively correlated to the sustainable development of the society (harmonious coexistence).

*H2c:* The degree of integration between film-induced culture and the tourism industry is positively related to the sustainable development of the economy.

Above all, we proposed the following effect hypothesis:

*H3a:* Film-induced tourism culture has a significant impact on sustainable development through integration degree;

*H3b:* Film-induced tourism industry has a significant impact on sustainable development through integration degree.

## Methodology

### Sample

To get a better and professional understanding of the dynamic mechanism of the culture and industry associated with film-induced tourism, the research subjects are limited to the management staff of the film-induced tourism industry. A total of 1,200 questionnaires were distributed, and 1,054 valid questionnaires were collected, with a recovery rate of 87.8%. The collected questionnaires were randomly divided into two equal sets (527 questionnaires in each set): one dataset is used for exploratory factor analysis and the other is used for confirmatory factor analysis. The demographic characteristics of the sample population are shown in Table 1. From Table 1 we can see, most of the responders are males (accounting for 68.9%), in the age groups of 25–35 and 36–45 (the total number of the two age groups accounting for 65.3%) and have a bachelor’s degree (accounting for 54.9%). 28% of the responders are tourism area managers; 34.7% of the responders are government department managers; and 37.3% of the responders are general staff. And the demographic characteristics of the responders generally follow the demographic distribution of the entire population in the area, indicating a good representativeness of the data and makes it an effective data source.

**Table 1:** Sample basic information.

Characteristic	Item	Percentage
Gender	Male	68.90%
	Female	31.10%
Age group	25 years and below	4.40%
	25–35 years	30.60%
	36–45 years	34.70%
	45–59 years	27.60%
	60 years and above	2.70%
Education	High school and below	10.40%
	Diploma	21.90%
	Bachelor	54.90%
	Master and above	12.80%
Employment	Tourism area manager	28.00%
	Government department manager	34.70%
	General staff	37.30%

### Measure

We draw on the mature scales used in previous studies for reference, and the initial scale was formed after corresponding modifications according to research topic. Then, two scholars who have been engaged in film-induced tourism and sustainable development were invited for analysis and discussion, and the scale was modified and improved. We use 5-level Likert scale to measure all variables, with 1 indicating “very unimportant” and 5 indicating “very important.” The specific measurement items and reliability are shown in the appendix. In addition, SPSS 26.0 was used for validity test, and KMO was 0.906 (>0.8). The results show that the scale has good reliability and validity, indicating that there is internal consistency among the variables (Table 2).

**Table 2:** Measurement items and reliability.

Constructs	Item dimension	Measurement items	Sources	Cronbach’s *α*
Film-induced tourism culture (FTC)	Content	The importance of cultural connotation to the degree of culture and tourism integration	McKercher, 2002; Gnoth and Zins, 2013	0.805
	Propagation	The importance of high-tech cultural communication media to the degree of culture and tourism integration		
Film-induced tourism industry (FTI)	Marketing	The influence of market competitive advantage on the development of cultural tourism	Atilgan et al., 2003; Yu et al., 2011	0.862
	Related Service	The influence of the intellectual support of all social parties on the development of cultural tourism		
Degree of Integration (DOI)	Integrated Resource-based View (IRBV)	The importance of linkage and integration of dynamic resources and industries to the development of film-induced tourism	Clark and Chabrel, 2007	0.792
	Integrated Ecology-based View (IEBV)	The importance of film-induced tourism industry to adapt to the environment		
	Integrated Space-based View (ISBV)	The importance of immersive film/television space for the development of film-induced tourism		
Sustainable development of Film-induced industry (SD)	Economic Sustainability (ES)	The impact of the rapid and healthy development of national economy on the sustainable development of film-induced tourism	Choi and Sirakaya, 2005; Kristjánsdóttir et al., 2018	0.877
	Human–environment Coordination (HEC)	The impact of effective utilization of film/television resources on the development of film-induced tourism		
	Harmonious Coexistence (HC)	The importance of harmonious coexistence between human and nature for the development of film-induced tourism		

## Results

### Confirmatory Factor Analysis

Confirmatory Factor Analysis (CFA) showed (Table 3) that P<0.01, and the Composite Reliability of all variables was 0.622–0.865, so that the polymerization validity and the convergence validity is good. AVE was in a reasonable range. Therefore, the results of CFA all meet the standard, and all dimensions have good convergence validity.

**Table 3:** Confirmatory factor analysis.

Construct	Item	*p*	*STD.*	*SMC*	*CR*	*AVE*
FTC	Content		0.856	0.733	0.806	0.676
	Propagation	0.000	0.787	0.619		
FTI	Attraction		0.817	0.667	0.865	0.681
	Marketing	0.000	0.828	0.686		
	Service	0.000	0.830	0.688		
DOI	IRBV		0.620	0.385	0.622	0.355
	IEBV	0.000	0.565	0.320		
	ISBV	0.000	0.601	0.361		
SD	ES		0.697	0.486	0.860	0.674
	HEC	0.000	0.885	0.782		
	HC	0.000	0.868	0.754		

FTC, film-induced tourism culture; FTI, film-induced tourism industry; DOI, degree of integration; SD, sustainable development of film-induced industry; IRBV, integrated resource-based view; IEBV, integrated ecology-based view; ISBV, integrated space-based view; ES, economic sustainability; HEC, human–environment coordination; HC, harmonious coexistence.

### Correlation Analysis

Through the correlation coefficient test, it can be seen that the values below the diagonal are, respectively, the correlation coefficients between potential variables (Table 4). Each potential variable has different connotations in theory, and each variable has relatively high correlation and good discriminant validity.

**Table 4:** Correlation analysis.

	Mean	SD	FTC	FTI	DOI	SD
FTC	4.16	0.692	1.000			
FTI	4.14	0.726	0.605^**^	1.000		
DOI	4.25	0.657	0.62^**^	0.520^**^	1.000	
SD	4.08	0.682	0.59^**^	0.643^**^	0.539^**^	1.000

FTC, film-induced tourism culture; FTI, film-induced tourism industry, DOI, degree of integration; SD, sustainable development of film-induced industry. **p < 0.05, **p < 0.01, ***p < 0.001.

### Goodness of Fit of the Structural Model

Based on the previous research results, the path relationship diagram between potential variables and observed variables has been built, the goodness of fit of the model to be verified have been tested from AMOS 26.0. The main fitting indicators all meet the ideal standard, that is, the model fitting effect is ideal.

### Hypothesis Testing

**Figure 1:**
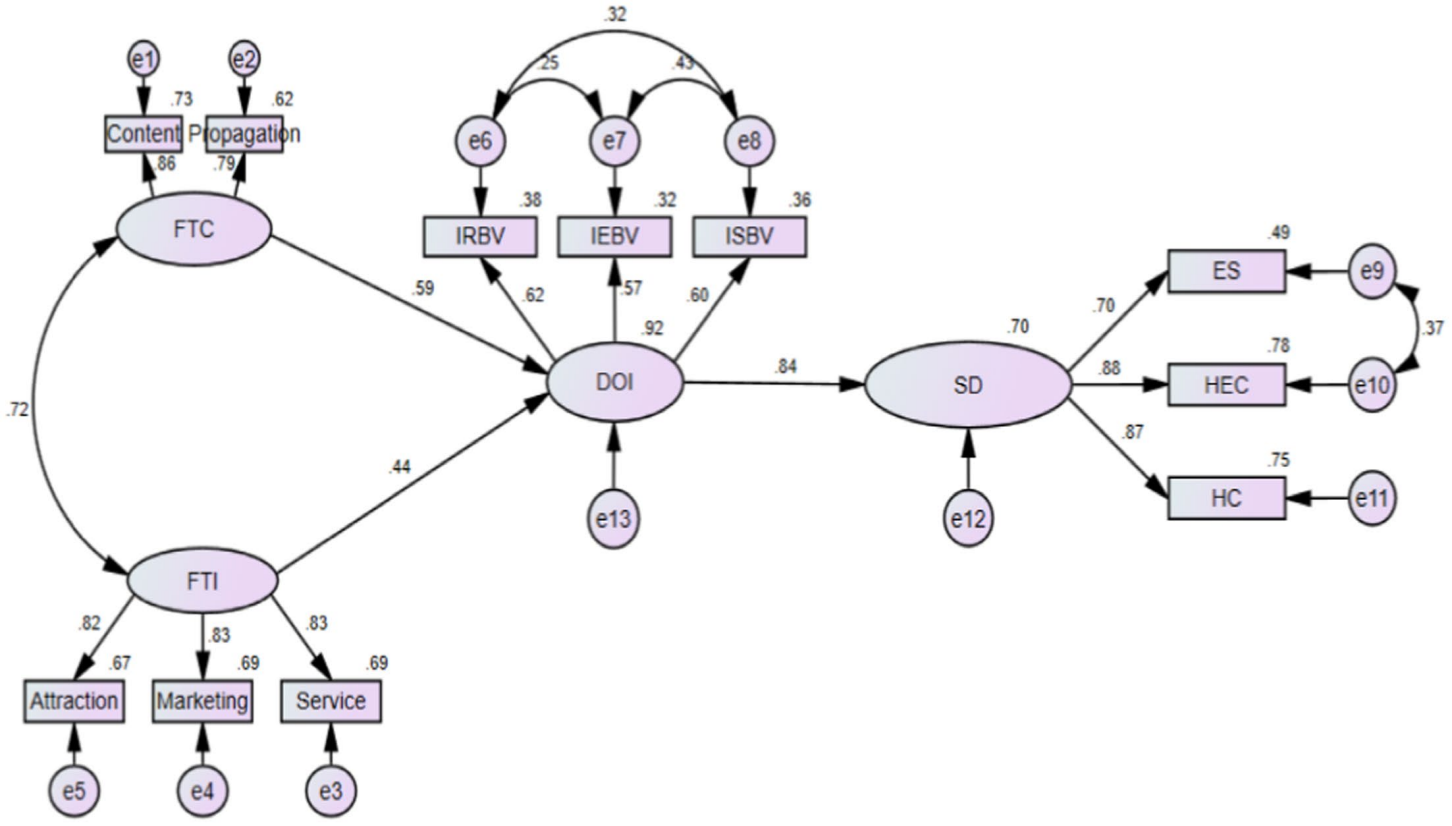
Structural equation model. FTC, film-induced tourism culture; FTI: film-induced tourism industry; DOI, degree of integration; SD, sustainable development of film-induced industry; IRBV, integrated resource-based view; IEBV, integrated ecology-based view; ISBV, integrated space-based view; ES, economic sustainability; HEC, human–environment coordination; HC, harmonious coexistence.

In order to further test the hypothesis proposed above, we run a structural equation model with mediation (see Figure 1). The results are shown in Table 5. There is a correlation between film-induced culture and film-induced industry (*r* = 0.720). Film-induced culture has a significant impact on the degree of integration (*r* = 0.590, C*R* = 7.495, *p* < 0.01); The film-induced tourism industry has a significant impact on the degree of integration (*r* = 0.441, C*R* = 6.326, *p* < 0.01), then hypothesis 1a, 1b, 1c are supported. The degree of integration has a significant positive impact on film-induced tourism (*r* = 0.836, C*R* = 11.817, *p* < 0.01), so hypothesis 2a, 2B, and 2C are also supported.

**Table 5:** Goodness of fit of the structural model.

Fitting index	Suggested value	Actual value	Fitting effect
CMIN/DF	1–3	2.019	ideal
GFI	>0.9	0.977	ideal
RMSEA	<0.05	0.043	ideal
CFI	>0.9	0.990	ideal
IFI	>0.9	0.990	ideal
TLI	>0.9	0.984	ideal
PGFI	>0.5	0.533	ideal
PCFI	>0.5	0.648	ideal
PNFI	>0.5	0.641	ideal

### Empirical Testing of Mediating Effects

In order to test the reliability of the path hypothesis, we further use Bootstrapping to calculate the mediating effect of culture and tourism integration. Bootstrapping test performs 3,000 samplings and selects a 95% confidence interval, then the final test results are shown in Table 6, so both hypothesis H3a and H3b are assumed to hold (Table 7).

**Table 6:** Hypothesis testing.

DV	IV	Un std.	SE	CR	p	Std.	Result
DOI	FTC	0.500	0.067	7.495	^***^	0.590	Pass
	FTI	0.335	0.050	6.726	^***^	0.441	Pass
SD	DOI	0.828	0.156	11.817	^***^	0.836	Pass

FTC, film-induced tourism culture; FTI, film-induced tourism industry; DOI, degree of integration; SD, sustainable development of film-induced industry. *p < 0.05, **p < 0.01, ***p < 0.001.

**Table 7:** Empirical testing of mediating effects.

Path	Point Estimate	Coefficients		Bootstrap		
		SE	Value of *p*	Bias-corrected 95%	Percentile 95%	
				Lower	Upper	Lower
Overall effect	0.494	0.069	0.001	0.349	0.626	0.354
FTC → SD						
Indirect effect	0.494	0.069	0.001	0.349	0.626	0.354
FTC → DOI → SD						
Overall effect	0.369	0.078	0.000	0.228	0.537	0.221
FTI → SD						
Indirect effect	0.369	0.078	0.000	0.228	0.537	0.221
FTI → DOI → SD						

FTC, film-induced tourism culture; FTI, film-induced tourism industry; DOI, degree of integration; SD, sustainable development of film-induced industry.

## Discussion

### Conclusion

Given previous scholars’ studies on film-induced tourism (Ringle, 2018; Marafa et al., 2020), we assume that cultural tourism will significantly affect the dynamic mechanism of sustainable development. More specifically, we believe that the organic integration of film-induced culture and tourism industry will have a significant impact on economic, social, and ecological sustainable development, which are the three dimensions of sustainable development. The results generally support our hypothesis that we view culture and tourism as a systematic whole rather than separate them and that culture and tourism integration is not simply “culture + tourism” or “tourism + culture.” We also confirm that the degree of integration of film-induced culture and tourism industry plays an important mediate role in the sustainable development of film-induced tourism. Although culture and tourism seem to be combined with each other in contemporary society, they have not developed the sustainable development dynamics of products and services innovation, nor developed a systematic operation mechanism. Consequently, the integration of culture and tourism is not to mechanically copy the two independent elements, but the key lies in the functional replacement and format innovation, complementary advantages, and the optimal combination of industrial elements.

### Managerial Implications

As culture and tourism are two huge and complex systems, both have relatively mature management operation mechanism, working path, industrial rules, and industry norms, while they are currently characterized by high growth and rapid development (Richards, 2018; McKercher, 2020). Therefore, in constructing the path of the integration of culture and tourism to promote sustainable development, the resource characteristics, functional differences, and technological advantages of film-induced culture and tourism industry should be fully considered in theory, and their similarity and relevance should be taken into account. In practice, we should not only make full use of the location conditions, resource endowments, and social and economic systems of film-induced culture and tourism destinations, but constantly identify the intersection points of products, industries, and enterprises according to the changes in market demand, and construct diversified industrial integration modes according to local conditions to determine develop directions in the integration of resources, technology, market and products, and administrative management.

From the perspective of tourism industry, support the development of organization forms that meet the needs of film-induced tourism development. The integration of industry should finally be reflected in the integration of organizations. Although the fundamental dynamics for the development of film-induced tourism is the development of market demand (Connell, 2005), it needs enterprises to be discovered and satisfied to find business opportunities. We suggest relevant departments relax film-induced tourism business licensing, strengthen information services, support enterprises to explore new business areas, support various cooperation, and even merger and acquisitions. From the perspective of film-induced culture, enrich the cultural connotation of film-induced tourism. Film-induced tourism should not be equated with general implanted advertising or simply build film-induced bases, but should dig deeply into the cultural connotation of film-induced tourism, closely focus on the core theme of film/television works, and deeply develop “post-film products” related to tourism derivative industry, and then systematically integrate them to form a cross-industry and compound film-induced tourism industry chain (Young and Young, 2008; Fan and Yu, 2021). In order to promote sustainable development, we further suppose that we should strengthen the research on film-induced tourism and explore the development mode and regulations of film-induced tourism.

### Limitations and Future Directions

This study provides some enlightenments on the theoretical exploration and practical management of film-induced tourism. Inevitably, there are several limitations, which can be addressed in future studies. First, the verification of the hypotheses is through the empirical analysis of collected questionnaires, lacking the support of actual cases. This can be improved by case analysis in follow-up studies. Second, the degree of integration between culture and industry is measured and defined by their characteristics in this study. However, the integration may also be affected by their underlying relationship. Their spatial production characteristics are also valuable for further investigation. In summary, in this research, the dynamic mechanism for sustainable development of film-induced tourism has been investigated, and conclusions have been drawn. This topic, however, still requires in-depth follow-up investigations from the research community.

## Data Availability Statement

The original contributions presented in the study are included in the article/supplementary material, further inquiries can be directed to the corresponding author.

## Ethics Statement

The studies involving human participants were reviewed and approved by School of Economics and Management, East China Jiaotong University, Nanchang, China. The patients/participants provided their written informed consent to participate in this study. Written informed consent was obtained from the individual(s) for the publication of any potentially identifiable images or data included in this article.

## Author Contributions

KY contributed to the empirical work, the analysis of the results, and the writing of the first draft. JinZ and JiaZ supported the total work of the KY. YZ and CX contributed to overall quality and supervision the part of literature organization and empirical work. RT contributed to developing research hypotheses and revised the overall manuscript. All authors discussed the results and commented on the manuscript. All authors contributed to the article and approved the submitted version.

## Funding

This project was supported by the General Project of the National Social Science Fund of China: Tracking Research on the Development of Western Urban Politics (20BZZ055), General project of Humanities and Social Sciences General Research Program of the Ministry of Education: Research on the Generation Mechanism and Resolution Path of “Fragmentation Phenomenon” of Urban Social Governance (19YJA810002), Social Science Planning General Project in Jiangxi Province (No. 21XW06), Jiangxi Province Culture and Art Science Planning General Project (No. YG2021087), and Jiangxi Province Colleges Humanities and Social Science Project (No. GL20214).

## Conflict of Interest

The authors declare that the research was conducted in the absence of any commercial or financial relationships that could be construed as a potential conflict of interest.

## Publisher’s Note

All claims expressed in this article are solely those of the authors and do not necessarily represent those of their affiliated organizations, or those of the publisher, the editors and the reviewers. Any product that may be evaluated in this article, or claim that may be made by its manufacturer, is not guaranteed or endorsed by the publisher.
